# Automated daily dose accumulation workflow for treatment quality assurance during online adaptive radiotherapy with a 0.35T MR‐linac

**DOI:** 10.1002/acm2.14594

**Published:** 2024-12-20

**Authors:** Mojtaba Behzadipour, Tianjun Ma, Rabten K. Datsang, Brandon Lee, Dane Pittock, Elisabeth Weiss, William Y. Song

**Affiliations:** ^1^ Department of Radiation Oncology Virginia Commonwealth University Richmond Virginia USA; ^2^ Clinical Science MIM Software Inc. Cleveland Ohio USA

**Keywords:** 0.35T MR‐Linac, automated adaptive dose accumulation, deformable image registration, inter‐fractional anatomical changes, MR‐guided adaptive radiotherapy

## Abstract

**Purpose:**

This study assesses a novel, automated dose accumulation process during MR‐guided online adaptive radiotherapy (MRgART) for prostate cancer, focusing on inter‐fractional anatomical changes and discrepancies between delivered and planned doses.

**Methods:**

A retrospective analysis was conducted on seven prostate cancer patients treated with a five‐fraction stereotactic body radiation therapy (SBRT), using a 0.35T MRIdian MR‐LINAC system. Daily plans were adapted when dose thresholds were exceeded. Planning MRI (pMRI) and daily MRIs (dMRIs) were imported into MIM software for automated and manual dose accumulation procedures. Rigid and deformable image registrations were followed by dose accumulation to compare delivered and planned doses. Manual and automated image registrations were compared by calculating the Hausdorff distance (HD), Jaccard, and DICE metrics.

**Results:**

Moderate discrepancies in dosimetric parameters for the planning target volume (PTV) were observed between auto‐accumulated and planned doses, such as $D_{95\%}$ and $D_{0.03\;\mathrm{cc}}$, with average differences of −0.60±0.61 Gy and −1.31±0.42 Gy, respectively. Volume differences of $V_{34.4\;\mathrm{Gy}}$ and $V_{36.25\;\mathrm{Gy}}$ indicated that auto‐accumulated doses consistently had lower numbers compared to planned doses, with mean discrepancies of −1.80%±1.05% and −2.82%±1.72%, respectively. Organs at risk (OAR) dosimetric parameters exhibited higher dose volumes in auto‐accumulated doses, with moderate differences (planned [cc] vs. auto‐accumulated [cc]) observed in parameters such as urethra PRV $V_{8.4\;\mathrm{Gy}}$ at (4.33±1.90vs.4.34±1.90), rectum $V_{24\;\mathrm{Gy}}$ at (1.17±1.53vs.1.74±1.91) and rectum $V_{28.2\;\mathrm{Gy}}$ at (0.38±0.55vs.0.59±0.71). The comparison between manually and auto‐accumulated doses revealed negligible variations, as also indicated by strong concordance in geometric indices and *t*‐test *p*‐values above 0.7.

**Conclusion:**

The automated workflow, developed in collaboration with MIM Software Inc., demonstrates high accuracy compared to manual accumulation. The moderate differences observed between planned and accumulated doses emphasize the need for accurate dose accumulation for adaptive plans.

## 1 INTRODUCTION

The rapid evolution of online image‐guided radiation therapy has led to a new era of unprecedented precision in the field of cancer treatment. This progress has fundamentally altered the methodology for delivering radiation doses to tumor volumes while concurrently minimizing harm to surrounding healthy tissues. During the course of treatment, patients may experience anatomical changes that can significantly influence dose distribution within their body, leading to deviations from the planned dose.^1^, ^2^ Accumulating the radiation dose received by critical tissue structures over the course of treatment is essential for comprehending the discrepancies between planned and delivered doses. This understanding is the foundation of medical decision‐making regarding the adaptation of treatment plans for future fractions based on the cumulative doses delivered in previous fractions. Through the accumulation of dose data from multiple treatment fractions, radiation oncologists can tailor therapy to individual patient's needs, ensuring the delivery of precise and personalized care.

Furthermore, dose accumulation plays a pivotal role in establishing a precise linkage between planned doses and clinical outcomes.^3^ This role gains importance in the evaluation of novel treatment modalities and the execution of trials within the domain of adaptive radiotherapy (ART). This vital concept enables clinicians to optimize treatment plans, evaluate treatment efficacy, and effectively manage potential side effects.

While dose accumulation holds promise in providing a more realistic representation of the delivered dose, it is essential to acknowledge the presence of uncertainties within the dose accumulation process, rendering the calculated dose distributions, at best, estimations of the delivered dose.^4^ Unavoidably, deformable image registration (DIR) is crucial in accurately calculating dose accumulation. Its ability to model complex anatomical changes, such as organ motion and deformation, is essential for achieving an accurate representation of cumulative radiation doses. The accuracy and fidelity of DIR directly influence the reliability of calculated dose accumulations, making it a foundational component in treatment planning and assessment.^3^

Currently, dose accumulation encompasses numerous research‐grade solutions.^5^, ^6^, ^7^, ^8^, ^9^, ^10^ However, few of these solutions have undergone comprehensive validation for clinical use or integration into commercial systems. Notably, none of these solutions have been specifically tailored and optimized for the unique challenges posed by online daily MR‐guided ART (MRgART).^3^ The imperative need for the development of a robust and precise dose accumulation solution tailored to the distinctive demands of MRgART remains a focal point of active research endeavors.

The accurate assessment of radiation dose in MRgART becomes more important as treatment adaptations become more frequent and precise. While the MR‐Linac solution provides invaluable real‐time imaging, its unique soft tissue contrast properties provide daily adaptive opportunities if any anatomical changes have been observed. However inter‐fractional anatomical and dose changes pose distinct challenges for dose accumulation and composite dose evaluation. To address the challenge, a specific adaptive platform within MIM (MIM Software, Inc.) has been developed in‐house, with MIM's collaboration, aiming to provide an accurate representation of the cumulative dose delivered to patients. Importantly, the MIM workflow performs the entire image registration process automatically, without user involvement in either the rigid or deformable registration steps.

Through comprehensive discussions and iterative refinement, we tailored this workflow to meet the unique requirements and demands of our clinical practice. More specifically, for the image registration step, the automated workflow focuses on planning target volume (PTV)_eval_—defined as the Planning Target Volume minus the overlap between the PTV and any OARs—with an additional margin of 3 cm in the anterior‐posterior and right‐left directions and 2 cm in the superior‐inferior direction. In this study, we performed a thorough evaluation of the in‐house developed adaptive dose accumulation platform with daily adaptive prostate cancer patients treated with an MR‐Linac. Planning MRI (pMRI) and daily MRI (dMRI) from seven patients, who underwent five‐fraction SBRT on the 0.35T MRIdian MR‐Linac system (ViewRay Systems, Inc.), were imported into MIM for overall performance evaluation.

## 2 METHODS AND MATERIALS

**FIGURE 1:**
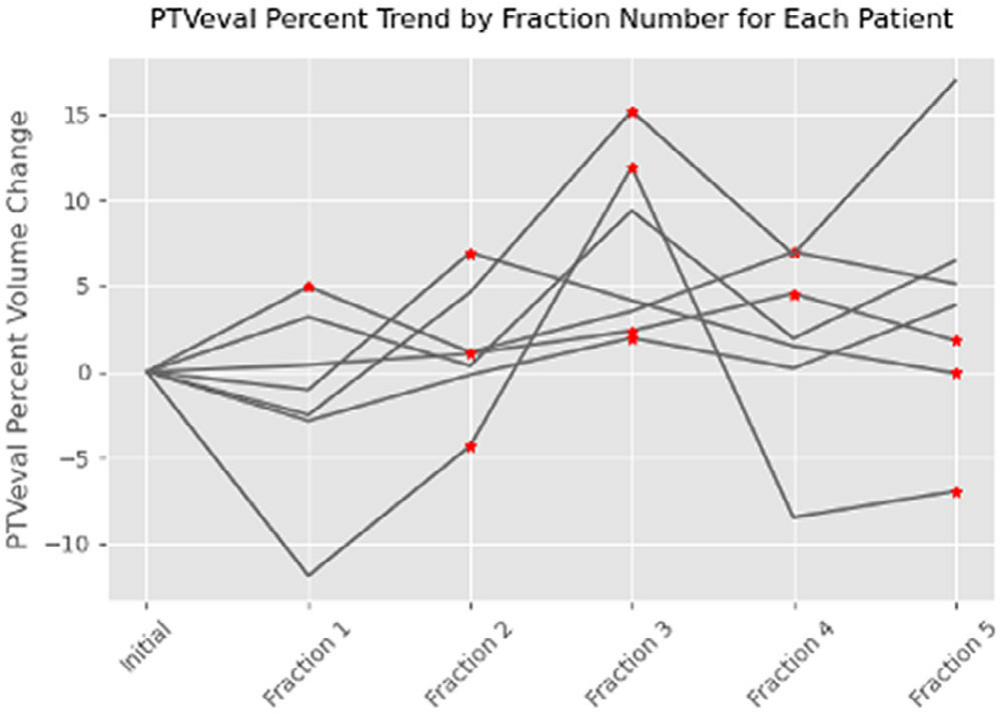
Inter‐fractional volume changes of PTV_eval_ for the seven patients. A red star at each fraction indicates that the covering physician differed from the primary physician. PTV, planning target volume.

Retrospectively, we conducted an analysis involving seven prostate cancer patients who had undergone treatment following institutional dose constraints (Table 1) using a 0.35T MRIdian MR‐LINAC system. These randomly selected patients underwent daily adaptive replanning, triggered whenever the target or OAR dose constraints deviated by more than ± 3%. For each fraction, new PTV_eval_ and OAR structures were contoured on the daily adaptive scan, and a new plan was generated to meet institutional criteria. This dose was then transferred to MIM and scaled back to one fraction in preparation for dose accumulation. Interfractional PTV_eval_ volume changes for these patients are shown in Figure 1. Patients received a five‐fraction adaptive SBRT regimen, with a prescription dose of 36.25 Gy delivered to 95% of the PTV_eval_ (7.25 Gy per fraction), and the average PTV_eval_ size from the initial plans for this cohort was 68.65 ± 37.69 cc.

**TABLE 1:** Institutional dose‐volume constraints for external beam treatment of prostate cancer.

PTV_eval_	Rectum	Bladder	Urethra PRV
≥ 95% at 36.25 Gy	≤ 0.03 cc at 38.06 Gy	≤ 0.03 cc at 38.06 Gy	≤ 0.03 cc at 38.78 Gy
≥ 98% at 34.4 Gy	≤ 3.00 cc at 34.40 Gy	≤ 10% at 18.12 Gy	**Femoral Heads**
≤ 20 cc at 38.78 Gy	≤ 10% at 32.60 Gy	**Penile Bulb**	≤ 10 cc at 19.90 Gy
≤ 0.03 cc at 43.50 Gy	≤ 20% at 29.00 Gy	≤ 0.03 cc at 36.25 Gy	
≥ 99% at 33.70 Gy	≤ 50% at 18.13 Gy	≤ 3 cc at 19.90 Gy	

Abbreviation: PTV, planning target volume.

**FIGURE 2:**
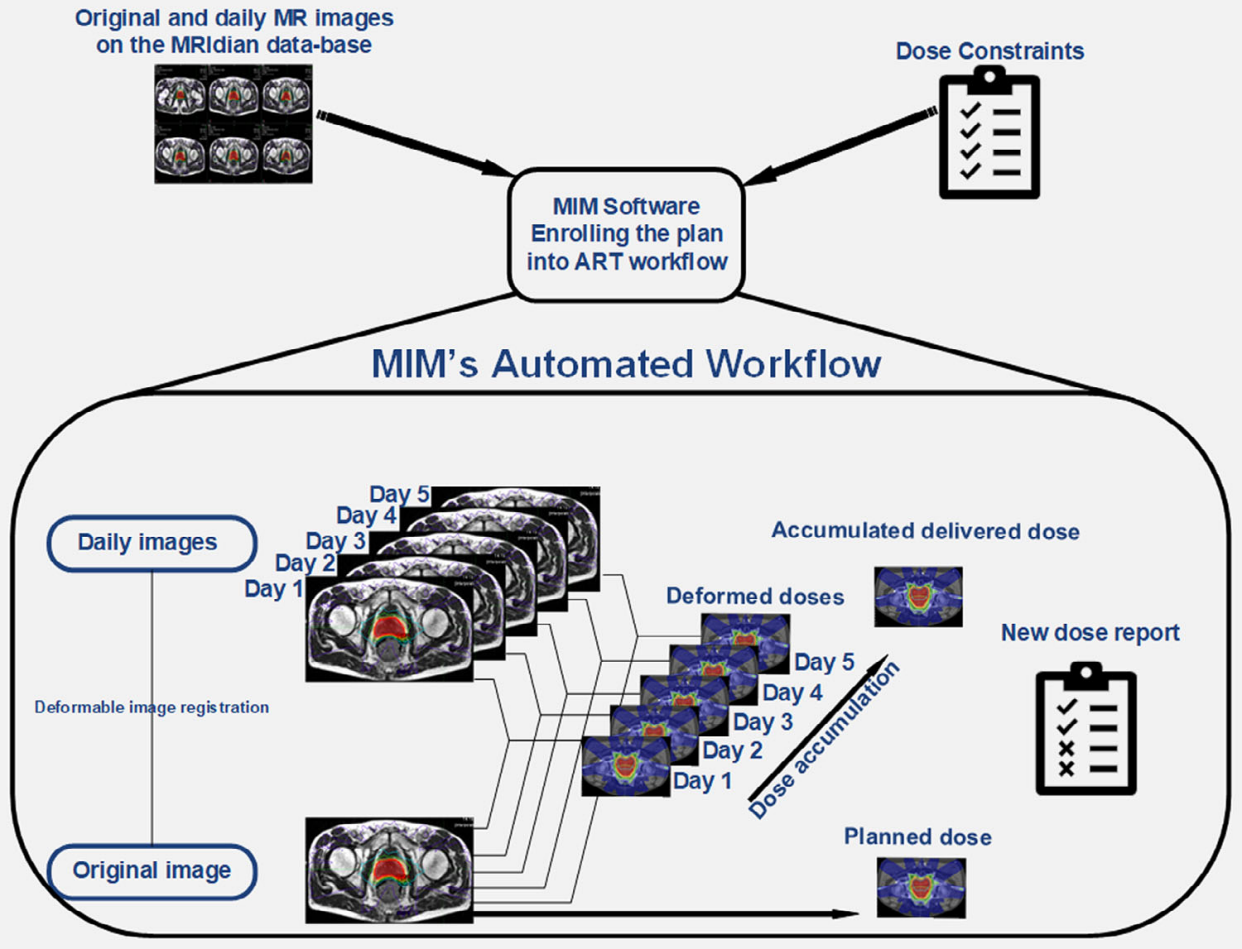
Dose auto‐accumulation workflow using MIM's software.

The original planning DICOM files, along with the daily adaptive DICOM files, were transferred into MIM, including DICOM RT plan, RT structures, and RT dose files. For dose reporting purposes, institutional dose‐volume constraints were created as a template within MIM's adaptive platform (ART Assist). MIM's adaptive platform automatically employs the pre‐selected workflow to help populate the accumulated delivered doses. For this study, 3D volumetric scans acquired with the true fast imaging with steady‐state free precession (TRUFI) sequence were used. TRUFI uses a T2/T1‐weighted contrast with 35 × 35 × 43 cm^3^ FOV and an isotropic spatial resolution of 0.15 cm. The custom‐built workflow first aligns the image via rigid image registration between the pMRI and dMRI. It then performs a hybrid DIR to align the PTV contours and generate the deformed daily fractional delivered doses back to the pMRI for progressive composite dose evaluation. Hybrid DIR is a combination of contour‐based and intensity‐based DIR, where the registration metric minimizes both the intensity differences between the two images and the differences between corresponding contour surfaces.^11^ The automated workflow completed the process in under a minute, for each treatment fraction. As a patient goes through each treatment fraction, the daily fractional delivered dose will gradually replace the planned dose fraction‐by‐fraction. In the end, an accumulated delivered dose will be generated to compare with the planned dose distribution as illustrated in Figure 2.

To validate MIM's automated adaptive dose accumulation procedure, we conducted a manual verification process, in which we manually performed rigid and then deformable image registrations between pMRI and each dMRI to enable the scaling and transfer of the delivered dose back to pMRI. The manual DIR was inspected and modified with landmark assistance if deemed necessary. To compare the manual and automated DIR processes, geometric metrics such as DICE, Jaccard, and HD were calculated for PTV_eval_ and the OARs.^12^ These metrics provided a quantitative assessment of the agreement between the two methods. In both the manual and automated workflows, the DIR process specifically focuses on the anatomical structures within a defined ring structure. This ring structure is used to prioritize the registration of key structures and target volumes, ensuring that alignment and deformation are optimized for these areas.

Eventually, all daily delivered doses were subsequently summed to derive the manually accumulated doses. The manual dose accumulation process took 8–15 min per fraction, with an average of about 10 min across all patients and fractions.

## 3 RESULTS

Geometric comparison of the PTV_eval_ and OARs between manual and auto‐accumulated doses demonstrated strong agreement. The DICE coefficient for the PTV_eval_ averaged 0.97 ± 0.02 across all patients, with similarly high agreement in the Jaccard index and low HD values. For OARs, the DICE values ranged from 0.86 to 0.97, again indicating close correspondence between the two methods. Jaccard index and HD values for the OARs further confirmed this strong geometric similarity. Table 2 presents the geometric indices calculated by comparing the manual and automated workflows for the PTV_eval_ and OAR contours across individual patients and the overall average across all patients.

**TABLE 2:** Geometric indices comparing manual and automated workflows for PTV_eval_ and OAR contours.

Anatomical site	Geometric indices	Patient 1	Patient 2	Patient 3	Patient 4	Patient 5	Patient 6	Patient 7	Overall averages
**PTV_eval_**	DICE	0.96 ± 0.03	0.98 ± 0.00	0.97 ± 0.01	0.95 ± 0.01	0.98 ± 0.00	0.95 ± 0.02	0.98 ± 0.01	**0.97 ± 0.02**
	Jaccard	0.92 ± 0.05	0.96 ± 0.01	0.94 ± 0.01	0.90 ± 0.02	0.97 ± 0.01	0.92 ± 0.04	0.96 ± 0.01	**0.94 ± 0.04**
	HD Max (mm)	2.16 ± 1.30	2.45 ± 0.89	2.43 ± 0.90	5.46 ± 1.52	1.98 ± 0.28	2.54 ± 1.04	1.87 ± 0.34	**2.70 ± 1.52**
	HD Mean (mm)	0.29 ± 0.17	0.28 ± 0.04	0.34 ± 0.08	0.43 ± 0.09	0.23 ± 0.06	0.37 ± 0.18	0.25 ± 0.06	**0.31 ± 0.13**
**Rectum**	DICE	0.96 ± 0.01	0.97 ± 0.00	0.97 ± 0.01	0.89 ± 0.03	0.95 ± 0.01	0.97 ± 0.01	0.94 ± 0.02	**0.95 ± 0.03**
	Jaccard	0.91 ± 0.02	0.95 ± 0.01	0.94 ± 0.02	0.81 ± 0.05	0.91 ± 0.02	0.95 ± 0.01	0.89 ± 0.03	**0.91 ± 0.05**
	HD Max (mm)	3.30 ± 0.86	1.90 ± 0.43	2.90 ± 1.25	8.58 ± 3.29	4.11 ± 1.44	2.29 ± 0.69	3.04 ± 1.54	**3.73 ± 2.64**
	HD Mean (mm)	0.34 ± 0.08	0.19 ± 0.02	0.23 ± 0.07	0.90 ± 0.27	0.32 ± 0.10	0.21 ± 0.05	0.30 ± 0.09	**0.35 ± 0.26**
**Right femur**	DICE	0.99 ± 0.00	0.98 ± 0.01	0.95 ± 0.01	0.97 ± 0.01	0.99 ± 0.00	0.92 ± 0.01	0.99 ± 0.00	**0.97 ± 0.03**
	Jaccard	0.97 ± 0.00	0.97 ± 0.01	0.89 ± 0.02	0.94 ± 0.03	0.97 ± 0.01	0.85 ± 0.02	0.97 ± 0.01	**0.94 ± 0.05**
	HD Max (mm)	1.67 ± 0.17	1.98 ± 0.53	14.11 ± 6.05	2.83 ± 1.00	1.70 ± 0.09	35.77 ± 3.17	1.67 ± 0.11	**8.53 ± 12.17**
	HD Mean (mm)	0.19 ± 0.01	0.21 ± 0.08	0.74 ± 0.19	0.36 ± 0.16	0.18 ± 0.04	1.73 ± 0.24	0.18 ± 0.04	**0.51 ± 0.55**
**Left femur**	DICE	0.99 ± 0.00	0.99 ± 0.00	0.95 ± 0.01	0.97 ± 0.01	0.99 ± 0.00	0.94 ± 0.01	0.98 ± 0.00	**0.97 ± 0.02**
	Jaccard	0.97 ± 0.00	0.97 ± 0.01	0.91 ± 0.02	0.95 ± 0.02	0.98 ± 0.01	0.88 ± 0.01	0.97 ± 0.01	**0.95 ± 0.04**
	HD Max (mm)	1.82 ± 0.17	1.69 ± 0.13	13.98 ± 5.93	2.40 ± 0.40	1.57 ± 0.02	25.42 ± 2.50	1.67 ± 0.17	**6.94 ± 8.97**
	HD Mean (mm)	0.18 ± 0.03	0.18 ± 0.05	0.65 ± 0.18	0.31 ± 0.12	0.13 ± 0.03	1.07 ± 0.17	0.23 ± 0.05	**0.39 ± 0.34**
**Penile bulb**	DICE	0.87 ± 0.10	0.88 ± 0.06	0.83 ± 0.29	0.87 ± 0.11	0.91 ± 0.05	0.96 ± 0.01	0.89 ± 0.04	**0.89 ± 0.13**
	Jaccard	0.78 ± 0.15	0.79 ± 0.10	0.80 ± 0.32	0.79 ± 0.15	0.85 ± 0.09	0.93 ± 0.02	0.80 ± 0.07	**0.82 ± 0.17**
	HD Max (mm)	1.48 ± 0.24	1.98 ± 0.67	2.63 ± 2.49	1.74 ± 0.37	1.76 ± 0.18	1.57 ± 0.02	1.59 ± 0.01	**1.82 ± 1.06**
	HD Mean (mm)	0.29 ± 0.18	0.37 ± 0.14	0.54 ± 0.86	0.32 ± 0.25	0.33 ± 0.16	0.23 ± 0.05	0.33 ± 0.09	**0.35 ± 0.37**
**Urethra PRV**	DICE	0.87 ± 0.06	0.89 ± 0.02	0.72 ± 0.10	0.89 ± 0.03	0.93 ± 0.03	0.82 ± 0.05	0.89 ± 0.03	**0.86 ± 0.08**
	Jaccard	0.77 ± 0.08	0.79 ± 0.04	0.58 ± 0.13	0.80 ± 0.05	0.87 ± 0.05	0.69 ± 0.07	0.81 ± 0.04	**0.76 ± 0.12**
	HD Max (mm)	3.32 ± 1.42	2.25 ± 0.47	1.91 ± 0.35	1.79 ± 0.38	1.89 ± 0.21	4.86 ± 2.11	1.85 ± 0.27	**2.56 ± 1.48**
	HD Mean (mm)	0.45 ± 0.21	0.34 ± 0.07	0.26 ± 0.10	0.29 ± 0.08	0.22 ± 0.08	0.45 ± 0.17	0.34 ± 0.08	**0.33 ± 0.15**
**Bladder**	DICE	0.96 ± 0.01	0.96 ± 0.02	0.96 ± 0.01	0.88 ± 0.06	0.97 ± 0.01	0.82 ± 0.12	0.96 ± 0.03	**0.93 ± 0.08**
	Jaccard	0.93 ± 0.02	0.93 ± 0.03	0.92 ± 0.02	0.79 ± 0.09	0.94 ± 0.02	0.72 ± 0.19	0.93 ± 0.05	**0.88 ± 0.12**
	HD Max (mm)	4.36 ± 1.26	3.51 ± 1.65	4.34 ± 1.79	10.78 ± 4.05	2.75 ± 0.95	9.58 ± 5.53	3.70 ± 3.40	**5.58 ± 4.29**
	HD Mean (mm)	0.41 ± 0.12	0.39 ± 0.17	0.47 ± 0.14	1.04 ± 0.43	0.32 ± 0.10	1.53 ± 1.04	0.41 ± 0.29	**0.65 ± 0.62**

*Note*: Since the column shows the overal results, the column is shown in bold font.

Abbreviations: OAR, organs at risk; PTV, planning target volume.

**FIGURE 3:**
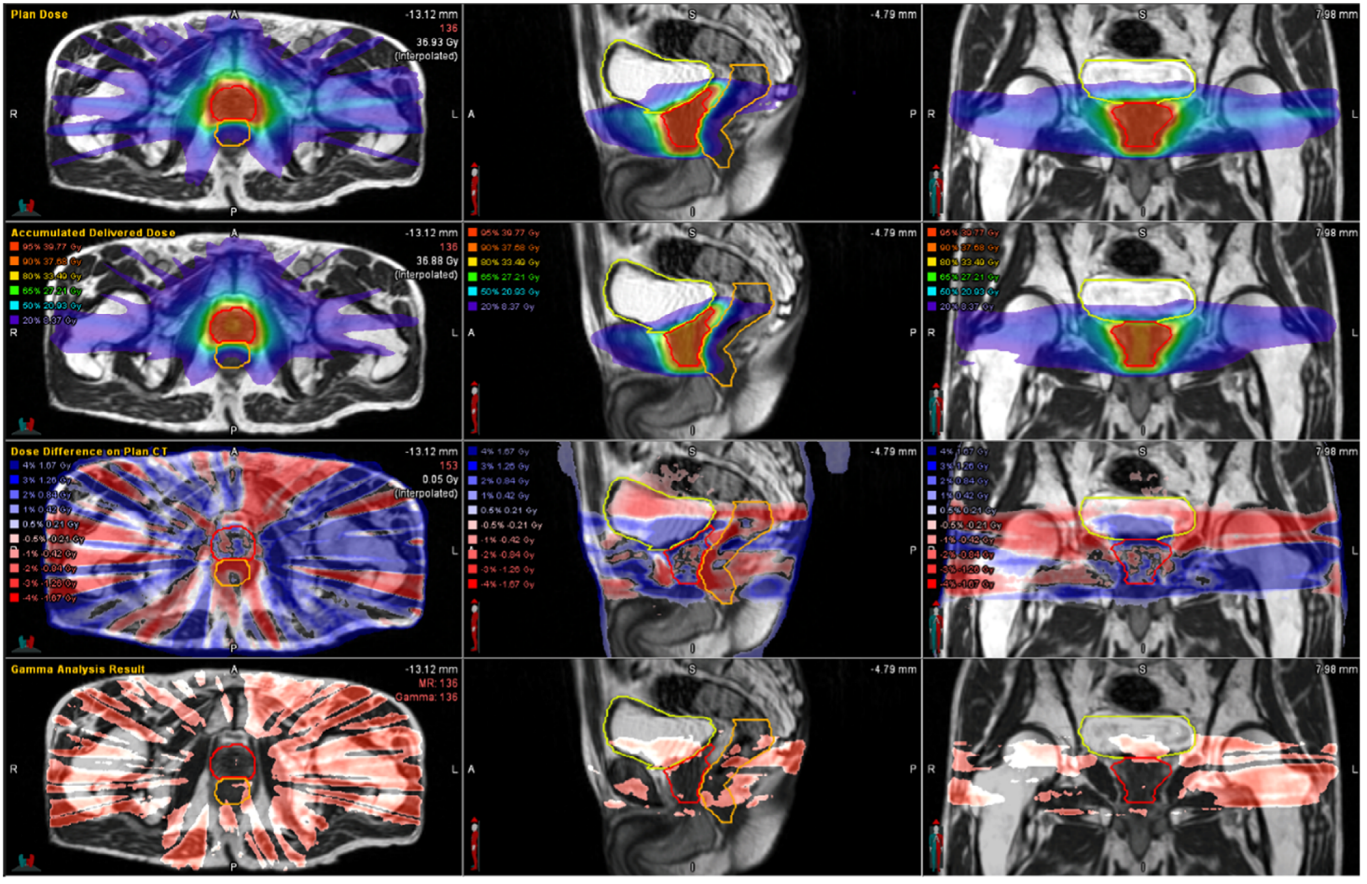
The difference between the original plan dose and the accumulated delivered dose. 1st row is the original plan dose, 2nd row is the accumulated delivered dose, 3rd row is the dose difference between the original plan dose and accumulated delivered. The 4th row shows the Gamma analysis results.

The results obtained through the automated dose accumulation workflow were compared with the planned doses. Figure 3 depicts the differences between the planned dose and the auto‐accumulated delivered dose for one patient.

**FIGURE 4:**
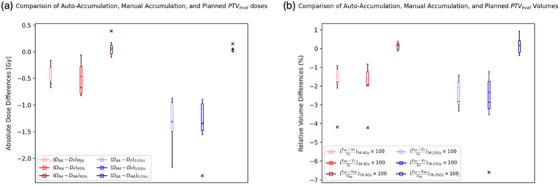
(a) Comparison of dosimetric parameters for the PTV_eval_ in the seven patients: red boxplots represent $D_{95\%},$ and blue ones represent $D_{0.03\mathrm{cc}}$. (b) Comparison of the percent volume discrepancy for the PTV_eval_: red boxplots represent $V_{34.4\mathrm{Gy}},$ and blue ones represent $V_{36.25\mathrm{Gy}}$. AA, auto‐accumulated; MA, manually accumulated; P, planned; PTV, planning target volume.

**FIGURE 5:**
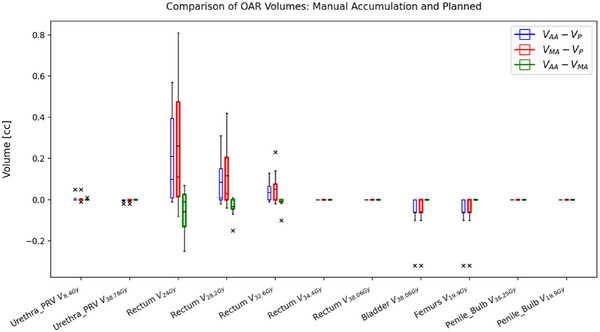
Comparison of certain dose volumes received by the OARs. OARs, organs at risk.

Dosimetric parameters, including $D_{95\%}$ and $D_{0.03\;\mathrm{cc}}$ for the PTV_eval_, were calculated for seven patients. These parameters were compared between auto‐accumulated and planned doses, manually accumulated and planned doses, and auto‐accumulated and manually accumulated doses, as illustrated in Figure 4a. Additionally, Figure 4b illustrates the percent volume discrepancy of certain doses delivered to the PTV_eval_ such as $V_{34.4\;\mathrm{Gy}}$ and $V_{36.25\;\mathrm{Gy}}.$ These discrepancies are calculated as $\left(\left(V_{\mathrm{Auto}-\mathrm{Accum}}-\;V_{\mathrm{Planned}}\right)\times 100/V_{\mathrm{Planned}}\right)$ between the auto‐accumulated and planned doses, the manually accumulated and planned doses, and the auto‐accumulated and manually accumulated doses. Figure 5 compares specific dose volumes received by the OARs between the auto‐accumulated and planned doses (blue), as well as between the manually accumulated and planned doses (red), and between the auto‐accumulated and manually accumulated doses (green). The dosimetric parameters used for these comparisons include urethra PRV $V_{8.4\;\mathrm{Gy}}$, urethra PRV $V_{38.78\;\mathrm{Gy}}$, rectum $V_{24\;\mathrm{Gy}}$, rectum $V_{28.2\;\mathrm{Gy}}$, rectum $V_{32.6\;\mathrm{Gy}}$, rectum $V_{34.4\;\mathrm{Gy}}$, rectum $V_{38.06\;\mathrm{Gy}}$, bladder $V_{38.06\;\mathrm{Gy}}$, femurs $V_{19.9\;\mathrm{Gy}}$, penile bulb $V_{36.25\;\mathrm{Gy}}$, and penile bulb $V_{19.9\;\mathrm{Gy}}$.

To evaluate the statistical significance of the differences observed between auto‐accumulated and manually accumulated doses, a two‐sample *t*‐test was conducted. The *p*‐values for PTV_eval_ $D_{95\%},\;D_{0.03\;\mathrm{cc}}$, $V_{34.4\;\mathrm{Gy}}$, $V_{36.25\;\mathrm{Gy}}$, and for the rectum ${\;V}_{24\;\mathrm{Gy}}$, $V_{28.2\;\mathrm{Gy}},$ $V_{32.6\;\mathrm{Gy}}$, $V_{34.4\;\mathrm{Gy}}$ in comparing manual and auto‐accumulated doses were 0.823, 0.915, 0.996, 0.995, 0.939, 0.899, 0.780, 0.712, respectively.

## 4 DISCUSSION

Our study primarily focused on the accuracy of the automated dose accumulation procedure we have developed. The geometric indices calculated for the comparison between manual‐ and auto‐accumulated workflows provide further insight into the reliability of our automated process. Overall, high DICE values for PTV_eval_ and major OARs such as the rectum and bladder indicate strong spatial agreement between the two workflows, with minimal boundary deviations as evidenced by low HD_Max_ and HD_Mean_ values. Moderate discrepancies were observed in the penile bulb and urethra. These discrepancies are due to the small volume and complex anatomy of these structures, where even minor contouring deviations can significantly impact geometric parameters such as the DICE and Jaccard Index.

Upon comparing the planned doses with the accumulated doses, we observed certain trends in the dosimetric parameters for the PTV_eval_ and OARs. Notably, we found minor discrepancies in parameters such as $D_{95\%}$ and $D_{0.03\;\mathrm{cc}}$ for the PTV, indicating instances of tumor underdosing in the auto‐accumulated doses compared to the planned treatment. The percent volume differences for $V_{34.4\;\mathrm{Gy}}$ and $V_{36.25\;\mathrm{Gy}}$ for the PTV_eval_ showed that the accumulated doses consistently exhibited lower volumes compared to planned doses. The study also assessed dosimetric parameters for various OARs, including the urethra PRV, rectum, bladder, femur, and penile bulb. For these parameters, accumulated doses were compared with planned doses. In most cases, dose accumulation showed a higher OAR dose‐volume compared to the planned doses. While some minor variations were observed between auto‐ and manually accumulated doses, these differences were typically small and may be attributed to factors such as uncertainties in the DIR process. The resulting *p*‐values for various parameters, including $D_{95\%}$, $D_{0.03\;\mathrm{cc}}$, and several volume metrics for the rectum ranged from 0.71 to 0.995. These values, being well above the conventional threshold of 0.05, suggest that the observed differences between manual and auto‐accumulated doses lack statistical significance.

The limitation of this study is the relatively small cohort size, encompassing seven patients with 35 fractions. While these cases provide valuable insights, the limited sample size may restrict the generalizability of our findings outside of our institution's treatment protocols. Larger, multi‐institutional studies are needed to validate our results and to understand the broader implications for diverse patient populations and varied clinical scenarios.

Incorporating the concept of adaptive fractionation (AF) in the context of image‐guided adaptive radiotherapy (IGART) underscores the critical importance of precise and reliable dose accumulation methods. Accurate quantification of the dose delivered to both the tumor and adjacent OARs at the end of each treatment fraction is an essential component for informed decision‐making in subsequent fractions. The utilization of accumulated dose data and daily imaging, which capitalize on the varying distances between the target and OARs, enables a machine‐learning model to adaptively plan daily doses. Thus, our study's advancements in dose accumulation methods not only enhance the accuracy of current treatments but also pave the way for the clinical feasibility of AF in IGART/MRgART. Further development and facilitation of dose accumulation is a significant step forward in the evolution of AF and personalized radiotherapy.

## 5 CONCLUSION

This study focused on evaluating inter‐fractional anatomical changes and their impact on dose deviation between planned and delivered doses in MRgART for prostate cancer. Our findings revealed a high degree of accuracy in the automated workflow we have developed, closely matching the results from manual verification processes. Moderate discrepancies were observed in dosimetric parameters such as $D_{95\%}$, and $D_{0.03\;\mathrm{cc}}$ for the PTV_eval_, between planned and delivered doses, indicating potential underdosing in tumor regions. This highlights the necessity of continuous adaptation in treatment planning to address anatomical changes, where platforms such as MRIdian MR‐Linac would provide value.

## AUTHOR CONTRIBUTIONS

*Analysis and interpretation of data*: Mojtaba Behzadipour, Tianjun Ma, Rabten K. Datsang, Elisabeth Weiss and William Y. Song. *Conceptualization*: William Y. Song and Tianjun Ma. *Methodology*: Mojtaba Behzadipour, Tianjun Ma, Rabten K. Datsang, Brandon Lee, Dane Pittock, Elisabeth Weiss and William Y. Song. *Validation*: Mojtaba Behzadipour, Tianjun Ma, Rabten K. Datsang, Elisabeth Weiss and William Y. Song. *Visualization*: Mojtaba Behzadipour, Brandon Lee and Dane Pittock. *Drafting the work*: Mojtaba Behzadipour. *Revising the draft*: Mojtaba Behzadipour, Tianjun Ma, Rabten K. Datsang, Brandon Lee, Dane Pittock, Elisabeth Weiss, and William Y. Song. *Software development*: Brandon Lee and Dane Pittock. *Final approval of the version*: Mojtaba Behzadipour, Tianjun Ma, Rabten K. Datsang, Brandon Lee, Dane Pittock, Elisabeth Weiss, and William Y. Song.

## CONFLICT OF INTEREST STATEMENT

Brandon Lee and Dane Pittock are employees of MIM Software Inc.
